# Optimally Sharp Energy Filtering of Quantum Particles via Homogeneous Planar Inclusions

**DOI:** 10.1038/s41598-019-56793-1

**Published:** 2020-01-21

**Authors:** Constantinos Valagiannopoulos

**Affiliations:** Nazarbayev University, Department of Physics, KZ-010000 Nur-Sultan, Kazakhstan

**Keywords:** Matter waves and particle beams, Quantum optics, Nanocavities

## Abstract

Some of the most influential players from academia and industry have recently expressed concrete interest for quantum engineering applications, especially for new concepts in controlling and processing the quantum signals traveling into condensed matter. An important operation when manipulating particle beams behaving as matter waves concerns filtering with respect to their own energy; such an objective can be well-served by a single planar inclusion of specific size and texture embedded into suitable background. A large number of inclusion/host combinations from realistic materials are tried and the optimally sharp resonance regimes, which correspond to performance limits for such a simplistic structure, are carefully identified. These results may inspire efforts towards the generalization of the adopted approach and the translation of sophisticated inverse design techniques, already successfully implemented for nanophotonic setups, into quantum arena.

## Introduction

Quantum interactions of particle beams with crystalline matter are present in numerous and diverse effects with significant quantum engineering potentialities. One can indicatively refer to matter waves tunneling through arbitrary potential distributions that support complex interference schemes^1^ or controllable transitions between vibrational states, crucial in quantum sensing^2^. Importantly, proper coupling between impinging particles and materials has been utilized for emulation of exotic electronic properties in heterojunctions^3^ and led to the systematic formulation of cavity quantum electrodynamics illustrating fundamental aspects of measurement theory^4^.

All these inherently fascinating phenomena being vital in disruptive quantum applications, have recently attracted huge funding interest; indeed, along with 5G communications and Artificial Intelligence, is one of the technologies that presidents of both, countries and companies, love to cite^5^. In particular, impressive State initiatives have been taken^6^, echoing major investments from Google, IBM, Intel, Microsoft and other industry giants, that are expected to ignite extensive quantum engineering research efforts in the near future. Already, National Science Foundation (NSF) is efficiently supporting research teams in quest of suitable hybrid materials used in quantum signal processing and computing^7^ while US Army Research Office (ARO) is currently funding studies on growing of matter for noise suppression towards high fidelity qubit operations^8^. Finally, several Multidisciplinary University Research Initiatives (MURIs) are executed with main objective to reformulate the range of light-matter interactions for quantum optics and photonic configurations^9^.

There are various alternative media employed in setups hosting the aforementioned effects; more specifically, one can use from isolated elements (germanium, silicon, carbon etc) and semiconductors (arsenides, antimonides, tellurides etc) to arbitrary alloys, mixtures and heterojunctions^10^. Not only reliable measurements and computational simulation models for the effective parameters^11^ describing the transport of quantum particles^12^ are available, but also several fabrication processes can be implemented for constructing, measuring and testing the corresponding prototypes. In particular, simple chemical precipitation methods have been adopted towards the synthesis of sulfide nanocomposites in graphene oxide building solar cell devices^13^ while exfoliation approaches are followed in growing telluride multilayers supporting preferential scattering observable through transport measurements^14^.

In addition, the coupling between arsenide quantum dots and an external fiber-mirror-based microcavity can be investigated by using mostly self-assembly techniques^15^ while quantum interference between single photons heralded from two independent micro-ring resonators has been measured fully-integrated onto a monolithic silicon photonic chip^16^. Diamond-based quantum layouts like nanowires into polycrystalline diamond fabricated via top-down methods allow for large collection efficiency of emitted photons^17^; moreover, unprecedented coherence of atoms combined with the scalability of a solid-state platform has been achieved by creating suitable defects into diamond^18^. It should be noted that diamond nanophotonic structures are additionally used for efficient light collection in hybrid integration with other material subsystems^19^ but, mainly, as solid-state quantum sensors exploiting nitrogen defects^20^.

One of the most typical objectives in modeling a quantum setup is the efficient energy filtering of the incident matter waves, namely the fabrication of a planar structure letting the impinging particles pass only if they possess a specific amount of energy, otherwise it blocks them^21^. Over thirty years ago, solutions based on sequential tunneling exploiting the, well-known from Photonics, Fabry-Perot mechanism^22^ led to tunable wavelength-selective detectors^23^. Moreover, systematic efforts towards filter design based on matter waves interference into potential superlattices by manipulating the emerged resonances have been recorded^24^, with the obtained results being experimentally tested^25^. Since then, interference-based energy filters have been used to perform very accurate scanning tunneling spectroscopic measurements^26^ and to execute electron counting with high resolution and sensitivity^27^. Most significantly, similar potential distributions with increased selectivity have played the role of modules in interferometric experiments complementing the field of electron holography^28^ and controlling the quantum transport phenomena in nanoscale systems via time -dependent fields^29^. It should be finally remarked that energy filtering plays a crucial role in nanostructured thermoelectric generators^30^, where the output power can be further boosted with help from Fabry-Perot cavities^31^ or in coolers with increased coefficient of performance^32^. Similarly, response selectivity with respect to the impinging energy of the beams is important in the operation of magnetic tunnel junctions with band pass utilities^33^ and quantum Hall setups^34^ considering the electron-nuclear spin flip-flops in the parametric vicinity of interest.

In this work, our aim is to provide realistic quantum designs with sharp response that can work as energy filters for the impinging matter waves. The structure is the simplest possible: a planar homogeneous slab embedded into a suitable environment; however, our search for optimal sizes and material combinations is exhaustive. In particular, we try and test every single quantum medium from a long directory in the role of host or inclusion and we conclude to the best designs that are transparent to a specific level of incoming particles energy, while being opaque to all the others energies belonging to an extensive band around it. Several alternative optimized designs are presented whose selectivity scores constitute performance limits of the considered simplistic setup provided the list of available media; similar conclusions have been drawn for different type of inclusions serving alternative purposes^35^. Fabry-Perot resonances of different orders, in proportion to what is the level of selected energy and the used materials, are activated at each filter layout giving different inclusion thicknesses and oscillation frequencies. The selectivity robustness of the designs with respect to fabrication defects of the slab size or engineering flaws influencing the effective parameters of the media is also found substantial even though shifts at the centrally filtered energies are noted.

This study reports numerous highly selective setups that deploy actual quantum matter and thus provides the interested experimentalists with additional degrees of freedom in fabrication of sharp energy filters for quantum particles and their respective matter waves. This primitive library of optimal setups performing such a ubiquitous operation at each selected energy level can be also useful in integrated systems design with state-of-the-art quantum engineering applications of a huge range spanning from attosecond time resolution^36^ and all-optical particle acceleration^37^ to optimal field detection^38^ and quantum signal processing^39,40^.

## Results

### Proposed setup

**Figure 1:**
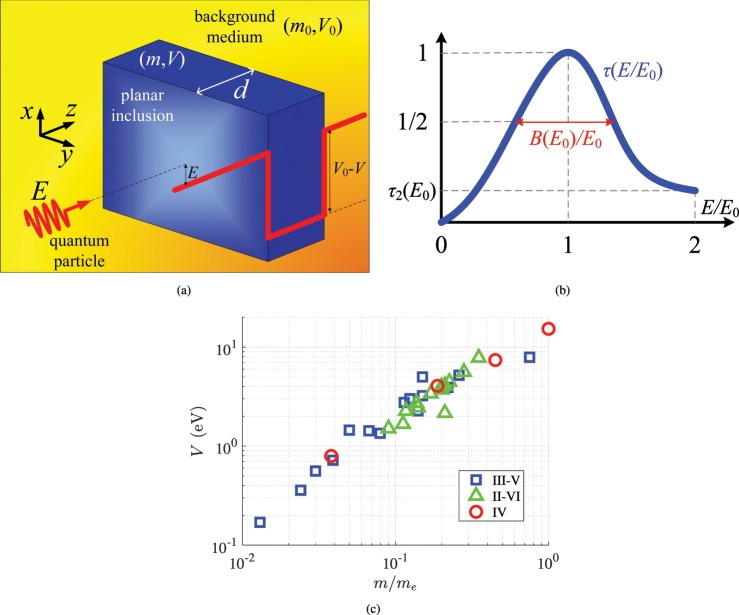
(**a**) The setup of the presented energy filter comprising a homogeneous planar inclusion with effective mass *m*, macroscopic potential energy *V* and thickness *d* into a specific background of effective parameters (*m*_0_, *V*_0_). The structure is excited by a normally incident electron beam of kinetic energy *E*. The considered structures correspond to quantum well configurations as shown in the schematic, namely to *V* < *V*_0_ . (b) Indicative variation of the transmissivity of the filter *τ*(*E*) for 0 < *E* < 2*E*_0_, where *E*_0_ is the selecting energy. The residual transmissivity *τ*_2_(*E*_0_) at *E* = 2*E*_0_ and the half-power energy bandwidth *B*(*E*_0_) are also defined. (**c**) Combinations of relative effective masses *m*/*m*_*e*_ and macroscopic potential energies *V* (in eV) of the materials populating our list used in the followed optimization.

We consider the physical configuration depicted in Fig. 1, where the employed Cartesian coordinate system (*x*, *y*, *z*) is also defined, comprising a planar inclusion of thickness *d* into a specific background, scattering a normally incident electron beam (e-beam) of energy *E*. The periodic crystals into the two regions excite Bloch waves following certain band structures affecting the particles moving within^41^; such an interaction is approximated by assuming that the motion of electrons is in free space with a different mass. In this way, the effective mass characterizing the travel into background is *m*_0_ and the corresponding quantity describing the particle trip into the cavity is denoted by *m*.

The macroscopic potential energy in each medium is defined as the minimal energy needed to extract an electron from the material into vacuum. It is taken spatially invariant into each of the two regions, namely the transition from the background (potential *V*_0_) to the filter (potential *V*) is assumed abrupt; note also that only the difference in the energy levels counts and thus we can use as reference the energy into the background (*V*_0_ ← 0). In this sense, we may take *V* ← *V* − *V*_0_ ≡ −Δ*V* and *E* ← *E* − *V*_0_, namely *E* will, from now on, denote the difference of the incoming particle energy from the host potential level. It should be also remarked that when considering the interface between two materials (like the ones normal to *z* axis), the potential-energy difference is not in general given by (*V* − *V*_0_). In particular, there is a charge redistribution across the boundaries, paving the way to bipolar charge development and causing an additional potential drop; such band offsets are neglected in the followed approach.

If the incident matter wave is described by the wave function Ψ_*inc*_ = exp(*ik*_0_*z*), the response of the slab from the other side is expressed by a similar matter wave with Ψ = *T*exp(*ik*_0_*z*), where ${k}_{0}=\sqrt{2{m}_{0}E}/\hslash$ is the wave vector norm into the background material and $\hslash$ is the reduced Planck constant. It is straightforward to find that the transmissivity *τ* = |*T*|^2^ is given by:

1 $$\tau =\frac{\mathrm{4(}{k}_{0}mk{m}_{0}{)}^{2}}{{({k}_{0}^{2}{m}^{2}+{k}^{2}{m}_{0}^{2})}^{2}-{({k}_{0}^{2}{m}^{2}-{k}^{2}{m}_{0}^{2})}^{2}{\cos }^{2}(kd)},$$

where $k=\sqrt{2m(E+\Delta V)}/\hslash$. Let us confine ourselves to quantum well configurations (*V*_0_ > *V* ⇒ Δ*V* > 0 ⇒ *k* > 0), as indicated in the schematic of Fig. 1, since we aim at observing interfering particles of arbitrarily small energy *E* higher than the potential level of background. By inspection of (1), it is clear that the transmissivity takes its maximal (unitary, *τ* = 1) value when cos(*kd*) = 1 ⇒ *kd* = *nπ* for *n* ∈ ℕ^*^, under the obvious constraint of *k* > 0. Accordingly, the quantity in (1) is minimized for cos(*kd*) = 0 ⇒ *kd* = *nπ* − *π*/2 (for positive integers $n\in {{\mathbb{N}}}^{\ast }$) with mi*n*imal values *τ* = [2*k*_0_*mkm*_0_/(*k*_0_^2^*m*^2^ + *k*^2^*m*_0_^2^)]^2^.

If one demands full transmissivity (*τ* = 1) at a single incoming particle energy level *E* = *E*_0_, defined by the application, it becomes feasible for specific sizes of the homogeneous planar inclusions: $d=n\pi \hslash /\sqrt{2m({E}_{0}+\Delta V)}$ for $n\in {{\mathbb{N}}}^{\ast }$. Our aim is to propose designs working as effective particle energy filters for a pre-determined energy across an *E*-range around the operational point *E* = *E*_0_. In particular, if we take this band to start from vanishing energies (*E* = 0), it is natural to assume a maximum level *E* = 2*E*_0_, symmetric with respect to *E* = *E*_0_. Needless to say that the followed approach can be also used for alternative functional ranges or multiple filtering energy levels. A typical graph *τ*(*E*) for 0 < *E* < 2*E*_0_ is illustratively depicted in Fig. 1, where the transmissivity gets trivially nullified for *E* = 0, it exhibits a single resonant peak at *E* = *E*_0_ with maximal value *τ* = 1 and decreases for *E*_0_ < *E* < 2*E*_0_; the half-of-maximum response *τ* = 1/2 is produced for two energies differing by *B*, which defines the resonance bandwidth. The merit of our filter is judged based on how suppressed is the response at the right extremum of the operational range *E* = 2*E*_0_ and how rapidly decays far from *E* = *E*_0_. With reference to Fig. 1, we are in search of designs possessing tiny residual transmissivities *τ*_2_ = *τ*(2*E*_0_) and as narrow half-power bands *B*(*E*_0_)/*E*_0_ as possible, at a given *E*_0_ each time.

### Optimal designs

This quest concerns an exhaustive search of all possible material combinations picked from a long list of quantum materials filling either the planar cavity^42^ or the background environment^10^, for a given level of selecting energy *E*_0_ that can vary within an interval 0.1 e*V* < *E*_0_ < 2 e*V*; once again, the range of *E*_0_ is just indicative and can be modified at will. The effective parameters of the used media are shown in Fig. 1(c), namely the combinations of effective masses *m* normalized by the inertial mass of electron *m*_*e*_ and their macroscopic potential energies *V* (in eV) are depicted on a plane. In our quest for optimal pairing of quantum media, we consider isolated semiconducting elements from group IV of the periodic table and various compounds between two elements of different groups (pairs of III and V or pairs of II and VI). Note that there is a proportional relation between the two represented quantities (*m*/*m*_*e*_, *V*) with the II-VI compounds possessing the middle values of both features, while the rest semiconductors cover much larger ranges. In this way, a coherent and extensive parametric area on (*m*/*m*_*e*_, *V*) map is occupied solely by regarding realistic and simple quantum texture, which can definitely serve as constituent in our optimization with respect to the effective quantities *m* and *V*. One major novelty of the present work is that these parameters are not taken as free continuous variables but correspond to actual quantum media and thus the proposed layouts, carrying certain beneficial characteristics, can be directly fabricated. As far as the evaluation of (*m*, *V*) is concerned, the potential energies *V* are derived through identifying the electronic energy-band structure for each material, where the quasi-cubic band model is employed^10^; on the other hand, the effective masses *m* are computed via estimating slopes of effective Hamiltonian for conduction and valence bands assisted from a theory of invariants^43^.

It should be stressed that the variation of *τ*(*E*) within a specified 0 < *E* < 2*E*_0_ is obviously more rapid when the thickness *d* becomes larger, namely the integer *n* gets higher as long as the condition *τ*(*E*_0_) = 1 is imposed^44^. For this reason, we have to select the integer *n* as big as possible but with care of retaining increasing transmission for 0 < *E* < *E*_0_ and decreasing for *E*_0_ < *E* < 2*E*_0_, otherwise queues from neighboring peaks will intrude into the considered energy interval 0 < *E* < 2*E*_0_. In Methods Section, it is shown that the optimal order of the occurred Fabry-Perot resonance is given by:

2 $$n=\lfloor \frac{\sqrt{{E}_{0}+\Delta V}\mathrm{/2}}{\sqrt{2{E}_{0}+\Delta V}-\sqrt{{E}_{0}+\Delta V}}\rfloor .$$

It should be also stressed that if we demand only selectivity around an energy level *E* = *E*_0_, without caring about what is happening across a much broader range (like 0 < *E* < 2*E*_0_), the obvious optimal resonance order is *n* → +∞. Indeed, for a giant *n*, which implies a huge thickness *d*, the response will be extremely sharp but with many other peaks at neighboring energies.

Some of the results from this trial-and-error process that correspond to a maximum *d* provided that a single resonance is “entrapped” into our parametric box, are shown in Table 1, where *E*_0_ = 0.1 e*V*; the rows of the Table refer to different background media while its columns indicate the employed quantum material of the planar inclusion. Only those cases that lead to sufficiently low *τ*_2_ are presented, meaning that numerous additional quantum texture combinations have been tested and rejected; note also that the best scores (in terms of *τ*_2_) are reached when the contrast between the potential energies of the two materials is substantial (${V}_{0}\gg V$). By inspection of Table 1, one observes extremely suppressed *τ*_2_ for most of the considered scenarios while the half-power bands *B* are tiny fractions of *E*_0_, designating very sharp filtering designs. There is a clear correlation between low *τ*_2_ and shrunk *B*/*E*_0_; importantly, the best filters are thicker, demanding slabs of several dozen of nanometers. Diamond, with its huge potential energy (*V*_0_ $\cong$ 15.3 e*V*), makes deep quantum well configurations and, when loaded by suitably-sized cavities, it creates ultra-performing quantum energy selectors. Indeed, diamond with certain nitrogen-based inclusions of significant stability in their electronic level structure has been additionally found to possess remarkable properties for quantum sensing applications^18,20^.

**Table 1:** Optimal combinations of planar inclusions media (columns) and background materials (rows) that block all the impinging quantum particles except those of energy *E*_0_ = 0.1 eV. The transmissivity *τ*_2_(*E*_0_) at *E* = 2*E*_0_, the half power energy band *B*(*E*_0_)/*E*_0_ and the optimal thickness *d* of the slab are shown in each box.

*E*_0_ = 0.1 eV	Indium Antimonide (InSb)	Indium Arsenide (InAs)	Indium Nitride (InN)	Gallium Antimonide (GaSb)	Cadmium Telluride (CdTe)
Silicon Carbide (SiC)	*τ*_2 _≅ 0.003 *B*/*E*_0_ ≅ 0.05 *d* ≅ 145.0 n*m*	*τ*_2_ ≅ 0.006 *B*/*E*_0_ ≅ 0.07 *d* ≅ 105.2 n*m*	*τ*_2_ ≅ 0.008 *B*/*E*_0_ ≅ 0.08 *d* ≅ 92.7 n*m*	*τ*_2_ ≅ 0.010 *B*/*E*_0_ ≅ 0.09 *d* ≅ 81.1 n*m*	*τ*_2_ ≅ 0.026 *B*/*E*_0_ ≅ 0.15 *d* ≅ 50.1 n*m*
Magnesium Oxide (MgO)	*τ*_2_ ≅ 0.004 *B*/*E*_0_ ≅ 0.06 *d* ≅ 149.0 n*m*	*τ*_2_ ≅ 0.007 *B*/*E*_0_ ≅ 0.08 *d* ≅ 108.1 n*m*	*τ*_2_ ≅ 0.010 *B*/*E*_0_ ≅ 0.09 *d* ≅ 95.4 n*m*	*τ*_2_ ≅ 0.012 *B*/*E*_0_ ≅ 0.10 *d* ≅ 83.4 n*m*	*τ*_2_ ≅ 0.031 *B*/*E*_0_ ≅ 0.16 *d* ≅ 51.8 n*m*
Boron Nitride (BN)	*τ*_2_ ≅ 0.017 *B*/*E*_0_ ≅ 0.04 *d* ≅ 149.9 n*m*	*τ*_2_ ≅ 0.003 *B*/*E*_0_ ≅ 0.05 *d* ≅ 108.8 n*m*	*τ*_2_ ≅ 0.004 *B*/*E*_0_ ≅ 0.06 *d* ≅ 96.1 n*m*	*τ*_2_ ≅ 0.006 *B*/*E*_0_ ≅ 0.07 *d* ≅ 84.0 n*m*	*τ*_2_ ≅ 0.014 *B*/*E*_0_ ≅ 0.11 *d* ≅ 52.2 n*m*
Diamond	*τ*_2_ ≅ 0.001 *B*/*E*_0_ ≅ 0.02 *d* ≅ 209.5 n*m*	*τ*_2_ ≅ 0.001 *B*/*E*_0_ ≅ 0.03 *d* ≅ 153.1 n*m*	*τ*_2_ ≅ 0.002 *B*/*E*_0_ ≅ 0.04 *d* ≅ 136.0 n*m*	*τ*_2_ ≅ 0.002 *B*/*E*_0_ ≅ 0.04 *d* ≅ 119.1 n*m*	*τ*_2_ ≅ 0.005 *B*/*E*_0_ ≅ 0.06 *d* ≅ 76.2 n*m*

In Table 2, we show the results of our optimization when the central filtering energy is tenfold higher: *E*_0_ = 1 e*V*. Obviously, the size of the structures is shrunk compared to the corresponding ones of Tables 1 since the propagating wavenumber in the background medium *k*_0_ is higher. Furthermore, the performance of the optimal designs is certainly lower as both the relative half-power bandwidth *B*/*E*_0_ and the residual transmissivity *τ*_2_ at *E* = 2*E*_0_ are much more substantial. The empty box indicates poor performance, namely *τ*_2_ climbs above 20% preventing the planar slab to work as an efficient energy quantum filter.

**Table 2:** Same as in Table 1 but for increased selecting energy *E*_0_ = 1 e*V*. Empty box indicates poor performance.

*E*_0_ = 1 eV	Indium Antimonide (InSb)	Indium Arsenide (InAs)	Indium Nitride (InN)	Gallium Antimonide (GaSb)	Cadmium Telluride (CdTe)
Silicon Carbide (SiC)	*τ*_2_ ≅ 0.03 *B*/*E*_0_ ≅ 0.16 *d* ≅ 15.0 n*m*	*τ*_2_ ≅ 0.05 *B*/*E*_0_ ≅ 0.21 *d* ≅ 11.2 n*m*	*τ*_2_ ≅ 0.06 *B*/*E*_0_ ≅ 0.23 *d* ≅ 10.1 n*m*	*τ*_2_ ≅ 0.08 *B*/*E*_0_ ≅ 0.30 *d* ≅ 7.8 n*m*	*τ*_2_ ≅ 0.18 *B*/*E*_0_ ≅ 0.45 *d* ≅ 5.5 n*m*
Magnesium Oxide (MgO)	*τ*_2_ ≅ 0.03 *B*/*E*_0_ ≅ 0.18 *d* ≅ 14.6 n*m*	*τ*_2_ ≅ 0.06 *B*/*E*_0_ ≅ 0.25 *d* ≅ 10.9 n*m*	*τ*_2_ ≅ 0.07 *B*/*E*_0_ ≅ 0.27 *d* ≅ 9.9 n*m*	*τ*_2_ ≅ 0.09 *B*/*E*_0_ ≅ 0.31 *d* ≅ 8.7 n*m*	
Boron Nitride (BN)	*τ*_2_ ≅ 0.02 *B*/*E*_0_ ≅ 0.12 *d* ≅ 14.6 n*m*	*τ*_2_ ≅ 0.03 *B*/*E*_0_ ≅ 0.17 *d* ≅ 10.8 n*m*	*τ*_2_ ≅ 0.03 *B*/*E*_0_ ≅ 0.19 *d* ≅ 9.8 n*m*	*τ*_2_ ≅ 0.04 *B*/*E*_0_ ≅ 0.21 *d* ≅ 8.7 n*m*	*τ*_2_ ≅ 0.11 *B*/*E*_0_ ≅ 0.35 *d* ≅ 5.3 n*m*
Diamond	*τ*_2_ ≅ 0.01 *B*/*E*_0_ ≅ 0.07 *d* ≅ 21.4 n*m*	*τ*_2_ ≅ 0.01 *B*/*E*_0_ ≅ 0.10 *d* ≅ 15.9 n*m*	*τ*_2_ ≅ 0.01 *B*/*E*_0_ ≅ 0.12 *d* ≅ 13.4 n*m*	*τ*_2_ ≅ 0.02 *B*/*E*_0_ ≅ 0.13 *d* ≅ 8.0 n*m*	*τ*_2_ ≅ 0.04 *B*/*E*_0_ ≅ 0.20 *d* ≅ 12.0 n*m*

**Figure 2:**
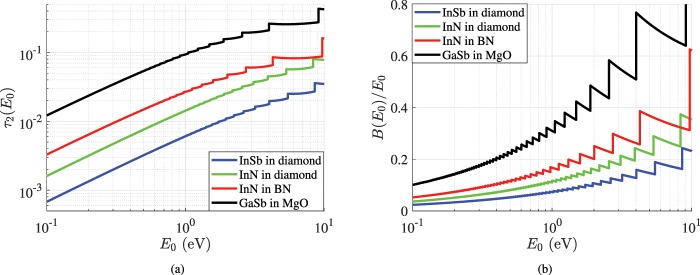
Optimal scores. (**a**) The residual transmissivity *τ*_2_(*E*_0_) at *E* = 2*E*_0_ and (**b**) The normalized half power energy bandwidth *B*(*E*_0_)/*E*_0_ of the resonances as functions of selecting energy *E*_0_, for several inclusion/background combinations optimized at every single level *E*_0_.

In Fig. 2, we select specific material combinations and represent the two basic figures of merit (*τ*_2_, *B*/*E*_0_) for all the considered amplitudes of filtering energies *E*_0_; in other words, the content of each box in Tables 1 and 2 correspond to a single point in the graphs of Fig. 2. In particular, Fig. 2(a) shows the variation of residual transmission *τ*_2_ with respect to operational energy *E*_0_ for four characteristic pairs of quantum media. There is a clear increasing trend of *τ*_2_ with *E*_0_ as also indicated by comparing Tables 1 and 2; it is thus demonstrated the much higher selectivity scores by such a simple setup when the energy of the propagating matter waves is low. One also directly notices the jumps of the curves at specific energy levels *E*_0_ which correspond to change of resonance orders *n* so that the appearance of a second peak within the considered energy range 0 < *E* < 2*E*_0_ is avoided. Note that the represented quantity exhibits stability between two successive discontinuities, while the jump turns larger when *E*_0_ gets more significant.

In Fig. 2(b), we show the relative half-power band *B*/*E*_0_ as function of *E*_0_ for the same texture combinations considered at Fig. 2(a). The selectivity of the filter certainly worsens for increasing *E*_0_ while the jumps to different resonance order *n* occur at the same *E*_0_-levels as in Fig. 2(a). Due to the waveform *τ*(*E*), and unlike with what is happening to *τ*_2_ at Fig. 2(a), the quality factor *B*/*E*_0_ of the filtering peak increases between two consecutive discontinuities. By inspection of Fig. 2(a,b), one can again understand the excellent job done by diamond as a host; the corresponding designs when loaded with dielectrics (InSb or InN) of specific thicknesses *d* work almost flawlessly as energy filters, especially at small magnitudes *E*_0_.

### Sharp energy filtering

**Figure 3:**
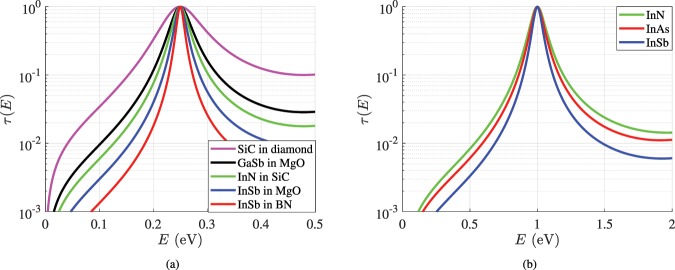
Transmissivity *τ*(*E*) as a function of the e-beam energy level *E* for (**a**) several optimal setups with *E*_0_ = 0.25 eV and (**b**) several optimal cavities hosted into diamond with *E*_0_ = 1 eV (included in Table 2).

It is meaningful to test the energy response for some of the best filter designs picked from Tables 1, 2 and Fig. 2. Therefore, in Fig. 3(a), we show the variation of *τ*(*E*) for several layouts with filtering energies of level *E*_0_ = 0.25 e*V*. We notice very rapid drops away from the central energy *E* = *E*_0_, given the fact that the vertical axis is logarithmic. One can find even better performing designs that are not examined in these set of results since our aim is to show the variety of material combinations. In fact, we have also considered a structure of moderate performance (SiC in diamond) not included in the Tables 1 and 2 (which, of course, are referring to different central energies) in order to show that a diamond host is not always enough for a super-selective output. Note also that the transmissivity vanishes for *E* = 0 ⇒ *k*_0_ = 0, as becomes obvious from (1).

In Fig. 3(b), we test three of the best designs from Table 2 (with *E*_0_ = 1 e*V*) recommending suitably grown slabs into diamond background and again the extremely sharp response of our reported setups is demonstrated. Our finding regarding the positive correlation of residual transmissivity *τ*_2_ and the half-power energy band *B*/*E*_0_ originating from graphs of Fig. 2 and Tables 1 and 2 is also verified; indeed, the more selective are the bell-shaped curves *τ*(*E*), the lower is the response at the right extremum *E* = 2*E*_0_ of the regarded energy interval 0 < *E* < 2*E*_0_. Finally, the positive influence of the contrast, either in terms of potential energies Δ*V* or regarding the effective masses *m*/*m*_0_, between the two utilized media on the filtering operation can be identified (InSb has a smaller potential *V* and a tinier effective mass *m* than the ones of InN).

**Figure 4:**
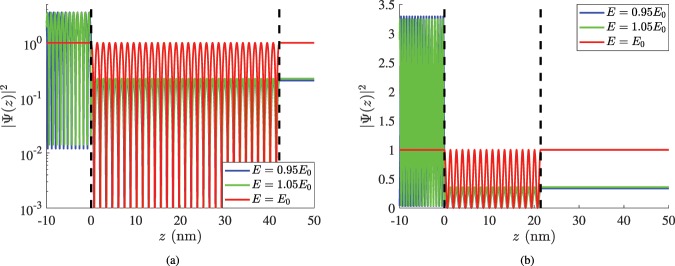
Spatial distribution of squared magnitude of the wave function |Ψ(*z*)|^2^ as a function of position *z* when the particle has energy exactly equal to the selected one (*E* = *E*_0_) and when *E* = *E*_0_(1 ± 5%) for InSb optimal planar inclusions into diamond with: (**a**) *E*_0_ = 0.5 eV, (**b**) *E*_0_ = 1 eV (included in Table 2). Black dashed lines denote the vertical boundaries of the filter.

Apart from the energy profile of the device response, it is also important to observe the variation of the wave function Ψ(*z*) across the propagation axis of the considered matter wave. In Fig. 4(a), we pick a specific optimal design (InSb planar inclusion into diamond with *E*_0_ = 0.5 e*V*) and evaluate the spatial distribution of squared magnitude |Ψ(*z*)|^2^ (which is proportional to the probability of finding the particle at position *z*) for three different impinging energies. When the filter is fed by an e-beam of energy exactly equal to the operational one (*E* = *E*_0_), the particles penetrate the cavity (whose boundaries are notated by dashed lines) with probability one. On the contrary, when the energy of the incoming matter waves is perturbed by only a small fraction of *E*_0_ (5%), the transmissivity drops substantially (to almost 10%) accompanied by sizeable reflections for *z* < 0. However, in all the three cases, the represented quantity oscillates many times within the planar slab (0 < *z* < *d*) which is the outcome of suitable matter waves interference in order for the desired behavior at *E* = *E*_0_ to be achieved. The principles behind this effect are identical to the ones giving photonic^22^ and quantum^23^ Fabry-Perot resonators. In Fig. 4(b), we examine the same material combination (InSb in diamond) as in Fig. 4(a) but optimized for different filtered energy *E*_0_ = 1 e*V*, namely has the size indicated by Table 2. As expected, the thickness is smaller since *k*_0_ is higher while the performance is deteriorated. Indeed, the transmissivities for *E* = *E*_0_(1 ± 5%) reach the quarter of its maximal value (25%); they are also almost equal each other (as happens in Fig. 4(a) too) revealing the locally quasi-symmetric nature of resonance around *E* = *E*_0_.

**Figure 5:**
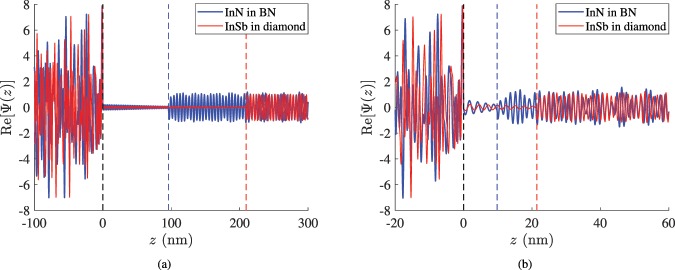
The quantum wave function signal Re[Ψ(*z*)] as a function of position *z* for a random mixture of various energies in two designs optimized for: (**a**) *E*_0_ = 0.1 eV and (**b**) *E*_0_ = 1 eV (included in Table 2), where the excitation includes a unitary matter wave operated at the optimal level of energy. Dashed lines denote the vertical boundaries of the filters (painted black for the common one at *z* = 0 and in the corresponding color for *z* = *d*).

It should be remarked that not only the magnitude of the wave function Ψ(*z*) has a physical meaning but also its real and imaginary parts are quantities employed in quantum signal processing^39,45^ and computing^46,47^. Therefore, in Fig. 5, we depict the signal Re[Ψ(*z*)] for two characteristic optimal designs as a function of position *z*. In Fig. 5(a), we consider a selected energy *E*_0_ = 0.1 e*V* (Table 1) and the structures are fed by an e-beam possessing random mixture of energies, where the optimal *E*_0_ participates with unitary magnitude. One can clearly observe that the chaotic input signal pattern for *z* < 0 is transformed into a harmonic output in both the considered designs propagating into the region right to the dashed boundaries of respective color (*z* > *d* in each case). Such a feature demonstrates the ability of the structure to let the matter waves of *E* = *E*_0_ pass, while blocking all the others with 0 < *E* < 2*E*_0_. The output signals are not identical; there are always some bi-products (unequal in the two scenarios) of small amplitudes corresponding to non-optimal incident energies.

In Fig. 5(b), we consider the same material combinations as in Fig. 5(a) but the structure is optimized for *E*_0_ = 1 e*V* (designs included in Table 2). Once again, the filtering operation exhibits a decreased efficiency compared to the cases working at smaller *E*_0_; however, the output of the devices still reminds us of a harmonic tone exp(*ik*_0_*z*) with specific spatial frequency *k*_0_ (different in the two graphs). Of course, the amplitude of the transmissive matter wave exhibits an envelop fluctuation being bigger in the less successful design that uses BN as host. Special mention should be made to the lower number of complete oscillations into the slab when the impinging energy *E*_0_ is more substantial, despite the fact that the operational wavelengths 2*π*/*k*_0_ of the incoming particles are smaller; the physical thickness *d* of the filter shrinks more. Note finally that the change of Re[Ψ(*z*)] signal into the planar filter resembles damped oscillations, indicating the asymmetry between the two oppositely propagating waves within the inclusion.

### Fabrication and engineering defects

As has been pointed out, there are several reliable fabrication methods of constructing quantum setups as the considered one in Fig. 1(a), namely creating in a specified host a planar cavity of certain thickness. The alternative approaches involve chemical characterization techniques combined with surface functionalization^13^ top-down lithographic fabrication^17^, plasma-sintering nano-structuring processes, or even self-assembled inclusions coupled with cavities^15^. Most of the aforementioned methodologies^19^ can give fine nanometer-sized^48^ structures; however, it is important to examine how differently the proposed filters are behaving under imperfect growing of sample thickness *d*. We also assume that the traveling of the matter wave into the background can be perfectly modeled, namely the parameters (*m*_0_, *V*_0_) and the energy *E* are exactly selected; accordingly, it would be interesting to investigate scenarios of wrongly estimated effective mass *m* or falsely engineered macroscopic potential *V* for the slab material.

**Figure 6:**
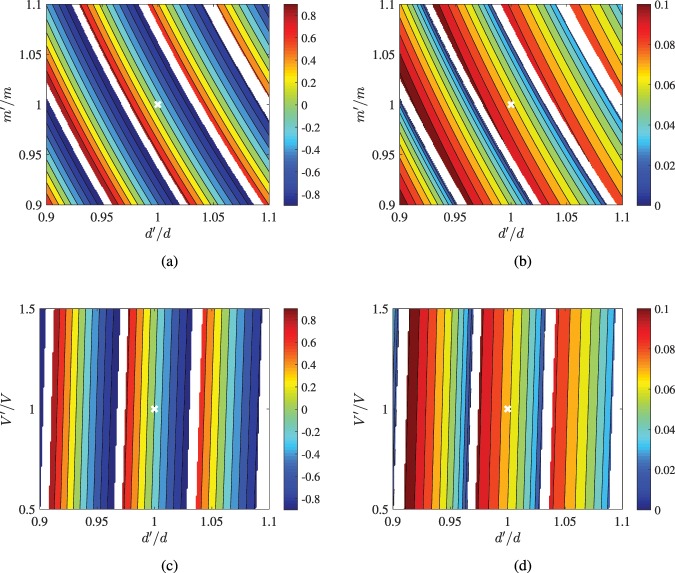
Performance sensitivity of the optimal planar inclusion of InSb into diamond for *E*_0_ = 1 e*V* (Table 2). (**a**) The relative difference in peak energy Δ*E*/*E*_0_ and (**b**) the half-power bandwidth *B* in eV as function of thickness misselection *d*′/*d* and effective mass misestimation *m*′/*m*. (**c**) The relative difference in peak energy Δ*E*/*E*_0_ and (**d**) the half-power bandwidth *B* in eV as function of thickness misselection *d*′/*d* and macroscopic potential energy engineering error *V*′/*V*. White regions corresponds to infeasible parametric combinations where our demand for a single peak of unitary transmissivity within the range 0 < *E* < 2*E*_0_, gets violated. White crosses denote optimal operational points.

In Fig. 6, we regard the most successful design of Table 2, namely the InSb planar inclusion into diamond (for *E*_0_ = 1 e*V*). We consider various thicknesses *d*′, effective masses *m*′ and potentials *V*′ around the optimal value *d* and the measured or simulated parameters (*m*, *V*) from standard textbooks^10^; wherever none or more than one *τ* = 1 peaks appear within the permissible interval 0 < *E* < 2*E*_0_, the design is labeled as infeasible and white color is used in the corresponding maps. Once such imperfections occur, the transmissivity maximum is usually observed at a different energy *E* = *E*′≠*E*_0_ and thus a quantity we selected to represent is the relative difference between the two peaks, denoted as (*E*′ − *E*_0_)/*E*_0_ = Δ*E*/*E*_0_.

In Fig. 6(a), we show this indicator Δ*E*/*E*_0_ as function of misselected thickness *d*′/*d* and incorrectly estimated effective mass *m*′/*m*. We observe several forbidden parametric regions followed by zones of feasible designs that form a rather periodic pattern with respect to both *d*′/*d* and *m*′/*m* as an outcome of the undamped oscillatory nature of the phenomenon. Indeed, we have lossless Fabry-Perot interference resulting to periodic waveforms for the device response as *d*′ is being swept; since infinite resonances (each one of a different order *n*), namely infinite slab sizes, give the desired *τ*(*E*_0_) = 1, the same happens for all the other values of transmissivity *τ*, which makes a periodic variation with respect to *d*′. Between two successive infeasible sections, large shifts of the peak transmission energy are recorded covering the entire working range 0 < *E*′ < 2*E*_0_; in particular, the energy *E*′ gets smaller than *E*_0_ for thinner slabs and greater effective masses. Note also that the boundaries between acceptable and non-acceptable parametric sets are not smooth due to the discrete nature of the resonances, similarly to Fig. 2; importantly, these boundaries are parallel to the smooth isocontours (into the colored region) demanding the size of the filters to decrease combined with a proper (almost linear) increase of the effective mass, to keep the represented quantity constant.

In Fig. 6(b) we represent across the same parametric plane (*d*′/*d*, *m*′/*m*) the half-power band *B* of the peak, expressed in dB. We notice that the infeasible regions are slightly more extended since we reject the designs whose range *E*′ − *B*/2 < *E* < *E*′ + *B*/2 does not fully belong in the permissible energy span; not just demanding 0 < *E*′ < 2*E*_0_ as in Fig. 6(a). One directly notices the small values of *B* since, in the worst (maximum *B*) case, the energy range is as short as 0.1 eV; such a feature demonstrates that the selectivity of our designs remains high under fabrication or engineering defects even though the peak occurs at another e-beam energy *E*′≠*E*_0_. Mostly surprisingly, the band *B*, within which the transmissivity falls at half, can be even better (smaller) than in the optimal design (for *E*′ = *E*_0_) yielding a sharper filtering. Such a finding does not question the followed optimization since it happens around very small energies $E^{\prime} \ll {E}_{0}$ where our best scores are much more substantial (like in Table 1).

In Fig. 6(c,d), we examine the effect of improper estimation for the macroscopic potential *V* of the planar inclusion, which is found mild given the fact that the isocontour lines are almost vertical. Such a feature is attributed to the huge potential of diamond *V*_0_ compared to that of InSb; whatever relative change it experiences, we always obtain ${V}_{0}\gg V$. In Fig. 6(c), where the difference Δ*E*/*E*_0_ is depicted, the trade-off between thicker designs and higher peak energies *E*′ is again noticed. Similarly, in Fig. 6(d), the half-power band *B* is larger when the peak energy *E*′ (at which *τ* = 1) increases, while keeping small values below 0.1 eV.

**Figure 7:**
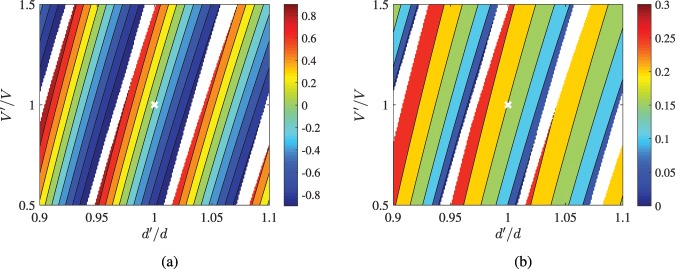
Similar calculations as in Fig. 7 but for the optimal planar inclusion of CdTe into diamond for *E*_0_ = 1 e*V* (Table 2). (**a**) The relative difference in peak energy Δ*E*/*E*_0_ and (**b**) the half-power bandwidth *B* in eV as function of thickness misselection *d*′/*d* and macroscopic potential energy engineering error *V*′/*V*.

In Fig. 7, we repeat the evaluations of the quantities of Fig. 6 but for a less efficient design from Table 1, namely CdTe into diamond; cadmium telluride is an isotropic noncentrosymmetric material with interesting properties exploited in photonic applications too^49^. The variations are similar with those of Fig. 6; the only difference is the wilder dependence of the represented quantities from the potential energy into the inclusion since *V* for CdTe is much higher than this into InSb. Finally, the values for the half-power energy band *B* are higher, a property related to the diminished performance of the considered layout as a filter.

## Discussion and Conclusions

Selecting matter waves with respect to the energy they carry is a significant operation in quantum signal processing, namely when e-beams are channeled, guided and funneled at different modules of an integrated quantum engineering setup. In this work, a simple configuration of a single planar inclusion into a dense background is used to filter an impinging particle beam via a maximally sharp transfer function. The optimal resonance order is determined by choosing the most abrupt variation without letting secondary maximum appear within a considered wide energy range. This process is meticulously repeated for a long list of quantum media combinations by using their effective properties. We show the results of our optimization both in tables and graphs for various energies and the positive correlation of the selectivity performance and the slab thinness with the operational level of energy has been identified. To this end, the functionality of certain designs is demonstrated by feeding them with particles of a variety of energies and observe them to practically block all the others except for the filtered ones. Finally, the device response is tested when the size and the texture of the inclusions are not properly estimated; it has been found that the energy at which unitary transmissivity is exhibited gets shifted but the designs remains selective.

This work reports the best scores of a simple, easy-to-fabricate setup given the set of available media; in this sense, presents limits in the selectivity of the configuration that cannot be surpassed unless a smarter structure (multilayers) or more complicated quantum texture (alloys, hybrid heterojunctions) is employed. An interesting next step would be to generalize our approach to find the best structure that mimics a given response at various distinct energies or even across a band of them. In this way, the purpose of inverse design will be served in quantum layouts. Thus, inspired approaches applied to nanophotonics involving sophisticated gradient optimizations^50^ or deep learning methods that have attracted the interest of both agenda-setting academic groups^51^ and industry research teams^52^, will be recasted and applied into quantum systems.

## Methods

The physical configuration of the device is depicted in Fig. 1(a), where a quantum particle traveling along an axis *z* with equal probability across each point of the formed normal *xy* plane, meets normally a homogeneous planar inclusion. The wave function Ψ(**r**) describing the probabilistic motion of the quantum particle into an arbitrary inhomogeneous medium with effective mass *m*(**r**) and potential *V*(**r**), respects the time-independent Schrödinger equation^53^:

3 $${\rm{\nabla }}[\frac{1}{m(r)}{\rm{\nabla }}]\Psi ({\bf{r}})+\frac{E-V({\bf{r}})}{{\hslash }^{2}/2}\Psi ({\bf{r}})=0,$$

where **r** is the related position vector. Our approach is semi-classical since the background medium and the considered inclusion is treated classically; only the impinging beam is assumed to exhibit quantum behavior. In addition, the effective quantum characteristics in each region are taken to vary abruptly without linear or other transition as one crosses the constant-*z* boundaries. The matter wave defining the behavior of the aforementioned particles in the absence of the slab inhomogeneity, possesses the form: Ψ_*inc*_(*z*) = exp(*ik*_0_*z*); however, in the presence of that thin cavity which is supposed to work as a quantum filter, the wave function for *z* < 0 changes by the reflecting-wave term Ψ_*ref*_(*z*) = *R* exp(−*ik*_0_*z*). In a similar manner, the entire matter wave behind the slab (for *z* > *d*) is given by: Ψ_*tran*_(*z*) = *T* exp(*ik*_0_*z*). As far as the wave function into the planar slab (0 < *z* < *d*) is concerned, it is written as: Ψ_*slab*_(*z*) = *C* exp(*ikz*) + *D* exp(−*ikz*). The followed technique is the well-known Wentzel-Kramers-Brillouin (WKB) approximation, where the wavefunctions are assumed to possess exponential forms with either amplitude or phase taken to be slowly changing relative to the de Broglie wavelength^54^.

The boundary conditions across the interface between two regions with effective masses {*m*_1_, *m*_2_} and wave functions {Ψ_1_, Ψ_2_} demand continuity of the probability amplitudes and the probability currents (**u** is the unitary vector normal to the boundary):

4 $${\Psi }_{1}={\Psi }_{2},\,\frac{1}{{m}_{1}}{\bf{\text{u}}}\cdot {\rm{\nabla }}{\Psi }_{1}=\frac{1}{{m}_{2}}{\bf{\text{u}}}\cdot {\rm{\nabla }}{\Psi }_{2}.$$

After imposing these requirements at *z* = 0, *d*, the unknown complex quantities {*C*, *D*, *R*, *T*} are determined; in particular, the reflection and the transmission coefficients are given by:

5 $$R=\frac{({k}_{0}^{2}{m}^{2}-{k}^{2}{m}_{0}^{2})\,\sin \,(kd)}{({k}_{0}^{2}{m}^{2}+{k}^{2}{m}_{0}^{2})\,\sin \,(kd)+2ik{k}_{0}m{m}_{0}\,\cos \,(kd)},\,T=-\frac{4\,\exp \,[id(k-{k}_{0})]k{k}_{0}m{m}_{0}}{\exp \,\mathrm{(2}ikd)({k}_{0}m-k{m}_{0}{)}^{2}-{({k}_{0}m+k{m}_{0})}^{2}}.$$

By assuming that effective masses {*m*_0_, *m*} and macroscopic potentials {*V*_0_, *V*} are selected so that *k*_0_, *k* > 0, one can evaluate the reflectivity *ρ* = |*R*|^2^ and the transmissivity *τ* = |*T*|^2^, which is shown in (1).

If one requests unitary transmissivity at a specific energy level *E* = *E*_0_, the thickness of the slab is permitted to take only discrete values $d=n\pi \hslash /\sqrt{2m({E}_{0}+\Delta V)}$ for $n\in {{\mathbb{N}}}^{\ast }$. By replacing the explicit forms for the wavenumbers ${k}_{0}=\sqrt{2{m}_{0}E}/\hslash$ and $k=\sqrt{2m(E+\Delta V)}/\hslash$, we obtain the following expressions:

6 $$\rho (E)=\frac{{[(1-m/{m}_{0})E+\Delta V]}^{2}{\sin }^{2}\,(n\pi \sqrt{\frac{E+\Delta V}{{E}_{0}+\Delta V}})}{4(m/{m}_{0})E(E+\Delta V)+{[(1-m/{m}_{0})E+\Delta V]}^{2}{\sin }^{2}(n\pi \sqrt{\frac{E+\Delta V}{{E}_{0}+\Delta V}})},$$

7 $$\tau (E)=\frac{4(m/{m}_{0})E(E+\Delta V)}{{[(1+m/{m}_{0})E+\Delta V]}^{2}-{[(1-m/{m}_{0})E+\Delta V]}^{2}{\cos }^{2}(n\pi \sqrt{\frac{E+\Delta V}{{E}_{0}+\Delta V}})},$$

where *n* is a positive integer.

The transmissivity (7), is maximized by taking unitary value (*τ* = 1) for $E+\Delta V=({E}_{0}+\Delta V){(\frac{\nu }{n})}^{2}$ with *ν* ∈ ℕ^*^; similarly, it gets minimized for $E+\Delta V=({E}_{0}+\Delta V){(\frac{\nu }{n}-\frac{1}{2n})}^{2}$ with minimum values *τ* = [2*k*_0_*mkm*_0_/(*k*_0_^2^*m*^2^ + *k*^2^*m*_0_^2^)]^2^. In this way, one can directly evaluate the optimal order *n* of Fabry-Perot resonance so that the energy response of the filter gets as sharp as possible; indeed, the first minimum of *τ*(*E*) when *E* > *E*_0_ occurs for *ν* = *n* + 1 and should be exhibited outside of the considered energy band 0 < *E* < 2*E*_0_. Namely:

8 $$({E}_{0}+\Delta V){(\frac{n+1}{n}-\frac{1}{2n})}^{2}-\Delta V > 2{E}_{0}\Rightarrow n < \frac{\sqrt{{E}_{0}+\Delta V}/2}{\sqrt{2{E}_{0}+\Delta V}-\sqrt{{E}_{0}+\Delta V}},$$

which is identical to (2). The residual transmission *τ*_2_ at the right extremum of the interval 0 < *E* < 2*E*_0_ can be also evaluated from (7) at *E* = 2*E*_0_ with use of (8).
